# Annotation of Peptide Structures Using SMILES and Other Chemical Codes–Practical Solutions

**DOI:** 10.3390/molecules22122075

**Published:** 2017-11-27

**Authors:** Piotr Minkiewicz, Anna Iwaniak, Małgorzata Darewicz

**Affiliations:** Department of Food Biochemistry, Faculty of Food Science, University of Warmia and Mazury in Olsztyn, Plac Cieszyński 1, 10-726 Olsztyn-Kortowo, Poland; ami@uwm.edu.pl (A.I.); darewicz@uwm.edu.pl (M.D.)

**Keywords:** peptides, glycopeptides, chemical modifications, bioinformatics, cheminformatics, chemical information, good practice, molecular editors, SMILES code

## Abstract

Contemporary peptide science exploits methods and tools of bioinformatics, and cheminformatics. These approaches use different languages to describe peptide structures—amino acid sequences and chemical codes (especially SMILES), respectively. The latter may be applied, e.g., in comparative studies involving structures and properties of peptides and peptidomimetics. Progress in peptide science “in silico” may be achieved via better communication between biologists and chemists, involving the translation of peptide representation from amino acid sequence into SMILES code. Recent recommendations concerning good practice in chemical information include careful verification of data and their annotation. This publication discusses the generation of SMILES representations of peptides using existing software. Construction of peptide structures containing unnatural and modified amino acids (with special attention paid on glycosylated peptides) is also included. Special attention is paid to the detection and correction of typical errors occurring in SMILES representations of peptides and their correction using molecular editors. Brief recommendations for training of staff working on peptide annotations, are discussed as well.

## 1. Introduction

Contemporary peptide science encompasses the biological and chemical approach. Medical sciences, pharmacology, biotechnology, and last, but not least, food and nutrition sciences need both biology and chemistry. Peptides are in the focus of interest of all the above-mentioned areas.

Tools for in silico peptide research, such as databases and programs, utilize both of the approaches that are classified as bioinformatics and cheminformatics, respectively, although most of the specialized databases and programs that are dedicated for peptides may be classified as bioinformatic tools [1]. Both of the approaches use different languages to describe the structures of biomolecules (e.g., peptides) [2,3]; bioinformatics operates based on amino acid sequences, whereas cheminformatics, on universal chemical codes. Communication between these two areas requires translation from the biological into the chemical language [2,3]. Dataset concerning biological activities of taste-affecting peptides, published in our review [4], may serve as an example of benefits from merging the biological and chemical approaches. It could not be completed without screening databases using peptide structures, annotated in the SMILES code [5,6], as a query. Another example of in silico research, utilizing chemical approach, has been recently published by Ortiz-Martinez and others [7]. They used the SwissTargetPrediction program [8,9], as provided by the Swiss Institute of Bioinformatics, Lausanne, Switzerland, to predict interactions between small peptides from maize and proteins of human organism. Chemical modifications of peptides, aimed to alter their biological activity, may recently be considered as a “hot topic” [10,11,12,13,14]. Processing of the peptide sequences, including non-protein or modified amino acids, is possible, e.g., using PepstrMod program [15,16] (provider Institute of Microbial Technology, Chandigargh, India). The above program utilizes hundreds of non-protein or modified amino acid residues. Possible space of non-natural or modified amino acids and other possible constituents of peptides, contains however even billions of possible molecules or molecule fragments [17]. SMILES and other chemical codes and formats enable the description of any artificially inserted substituents for in silico study of properties of modified peptides. Another approach is the search for peptidomimetics, which are potentially useful as drugs [18], on the basis of known peptide structures.

Many programs, utilizing the SMILES code, are recently available, including, e.g., BioTriangle program provided by the Central South University, Changsha, China [19,20], which serves to calculate, e.g., physicochemical and topological parameters of small molecules. Some programs that are utilizing SMILES are available via the website of the Swiss Institute of Bioinformatics, Lausanne, Switzerland [21]. This website offers access to, e.g., to SwissADME program [22,23] which allows predicting properties that affect substance applicability as a drug. Another example of program utilizing the chemical code is WebMolCS [24,25] and other programs developed at the University of Bern, Bern, Switzerland [26] within the Chemical Space Project [17]. Apart from the above, free accessible programs, there are also commercial tools, such as JChem or MadFast [27], both are provided by ChemAxon, Budapest, Hungary, utilizing chemical codes for database screening or calculations. There are also specialized peptide databases using the SMILES code, such as Brainpeps [28,29] or Quorumpeps [30,31], provided by the University of Ghent, Belgium; AHTPDB [32,33], CancerPPD [34,35], Hemolytik [36,37], ParaPep [38,39] or PepLife [40,41], provided by the Institute of Microbial Technology, Chandigargh, India. The above resources are integrated via the SATPdbmetabase [42,43]. Another example is BIOPEP database of sensory peptides and amino acids [44,45], provided by the University of Warmia and Mazury in Olsztyn, Poland. The number of programs and databases utilizing chemical codes successively increases. More links to such tools are available via metabases and metaservers [46,47,48,49,50].

The bioinformatics approach concerning peptides involves, e.g., modeling structures and predicting interactions with biomacromolecules on the basis of amino acid sequences [1,51]. Structure modeling, involving amino acid sequences, may be performed using programs such as PepstrMod [15,16], Pep-Fold [52,53] (provider: University of Paris, Diderot, Paris, France), or (PS)^2^.v3 (provider: National Chiao Tung University, Hsinchu, Taiwan) [54,55]. For instance, the Quantitative Structure-Activity Relationship (QSAR) approach involves a set of parameters that are describing the structure and physicochemical properties of particular amino acid residues [56,57]. The sequence-based approach is expanded using pseudo-amino acid composition [58,59,60]. The application of chemical information for annotation of peptides and for processing their structures may enlarge the array of tools available for peptide research in silico.

Cheminformatics tools cannot, however, be considered and used uncritically as ”black boxes”. Many published datasets contain errors. Users or curators of databases and programs using chemical information should be prepared to recognize and correct possible errors [61,62,63,64]. Validation of representations, identification, and correction of mislabeled compounds is recommended as one of the crucial steps of compound dataset preparation and curation [2,63]. The preparation of peptide datasets, involving translation from amino acid sequences into chemical codes, is not an exception. Peptide data, annotated using chemical information codes, requires careful inspection before use.

The aim of this review article is to present practical solutions concerning translation of peptide annotation from biological into chemical language and correction of possible errors using contemporary software (with special attention of non-commercial programs) without extensive historical background. Proposed recommendations are based on our experience with completion and curation of the BIOPEP database of sensory peptides and amino acids [44,45] and MetaComBio website (University of Warmia and Mazury in Olsztyn, Poland) [48,49].

## 2. Codes for Annotation of Peptide Sequences and Structures

### 2.1. Annotation of Peptides Using Biological Codes

The most common biological codes for the annotation of peptide sequences are: single letter code and multi-letter code. The first is applied for annotation of protein sequences and peptides consisting of amino acids present in proteins. Annotation of the peptides containing non-protein or non-natural amino acid residues requires a multi-letter code (usually three or four characters). The most comprehensive list of abbreviations of amino acid names may be found in the SwissSidechain (Swiss Institute of Bioinfomatics, Lausanne, Switzerland) [65,66] and Norine (University of Sciences and Technologies of Lille, Villeneuve d’Ascq, France) [67,68] databases. l-amino acids are annotated using capital letters, whereas d-amino acids—using small letters. Such a layout is used in, e.g., CycloPS program (University College Dublin, Ireland) [69,70], and the SATPdb database (Institute of Microbial Technology, Chandigargh, India) [42,43]. The last database annotates protein amino acids and their D-enantiomers using a single letter code, whereas non-protein amino acids—using a multi-letter code. Peptides containing amino acids from both groups are annotated using a “mixed code”, utilizing both single- and multi-letter amino acid abbreviations. The multi-letter symbols are divided by dashes. Amino acid symbols that are used in the SwissSidechain database do not include dashes within amino acid abbreviations. Such abbreviations may be sufficient to construct machine-readable sequences with the help of multi-letter and mixed codes. The Norine database utilizes symbols, including dashes. Amino acid sequences utilizing symbols containing dashes may be annotated using the LINUCS code, designed originally for the description of oligosaccharides [71]. LINUCS is also used to annotate peptide sequences in the PubChem database (National Center for Biotechnology Information, Bethesda, MD, USA) [72,73]. LINUCS code may offer machine-readable representation of glycopeptides. Another code applicable for this purpose is HELM [74,75], utilizing single letter symbols of amino acid residues and describing modifications at atomic level. HELM representations of peptides may be found e.g., in PubChem and ChEMBL (European Bioinformatics Institute, Hinxton, UK) [76,77] databases. Macrocyclic peptides may be annotated using amino acid sequences with SMILES rules for description of multiple rings [78].

### 2.2. Representation of Peptides Using SMILES Code

The most known chemical codes, which are used to annotate compound structures, are: SMILES [5,6], InChI [79], and InChIKey [79]. SMILES is the most popular chemical code used in databases of low molecular-weight compounds. It is applied in such databases as PubChem [72,73], ChemSpider (Royal Society of Chemistry, London, UK) [80,81], ChEMBL [76,77], or ZINC 15 (University of California San Francisco, CA, USA) [82,83]. The so-called isomeric SMILES, taking into account stereoisomers (e.g., configuration around asymmetric carbon atoms), is usually applied as a representation of chiral compounds, including amino acids and peptides. Databases and programs supporting mass spectrometric analysis may use the canonical SMILES (without the discrimination of configurations around asymmetric carbon atoms) due to the fact that mass spectrometry is unable to discriminate between stereoisomers. The canonical SMILES is applied to annotate peptides in, e.g., HMDB database (University of Alberta, Edmonton, AB, Canada) [84,85].

**Figure 1:**
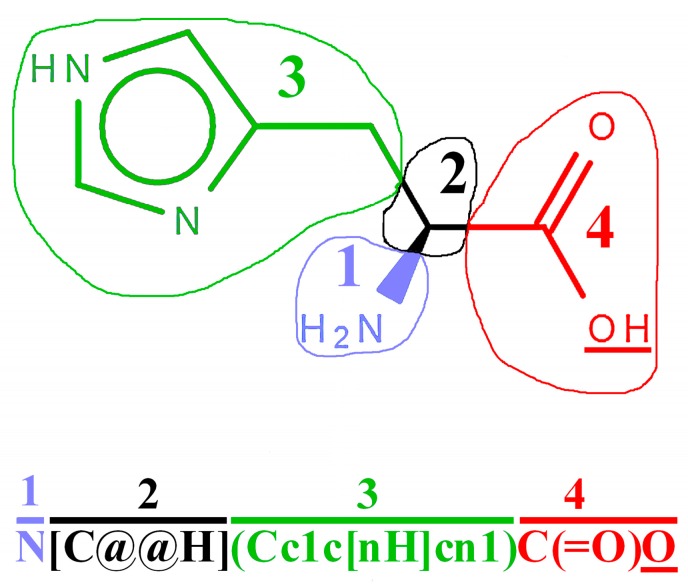
Construction of SMILES string of exemplary amino acid (L-histidine), sufficient for peptide annotation. (1) α-amine group; (2) carbon atom C2; (3) side chain (in this case methylene group and imidazole ring); and, (4) α-carboxyl group. Underlined hydroxyl group in the structure or oxygen atom in SMILES string is replaced by the next amino acid residue during construction of peptide representation. Figure prepared with the help of Ketcher 2.0 molecular editor (EPAM Systems, St. Petersburg, Russia) (Demo version) [86,87].

Multiple SMILES representations for the same compound are also possible. They may differ by, e.g., order of symbols indicating particular atoms [3,6]. The order of symbols, as proposed by Siani et al. [2], and presented in Figure 1, i.e., α-amine group; asymmetric carbon atom C2; side chain, and carboxyl group, is sufficient for the construction of SMILES representations of peptides. Such SMILES codes are presented in, e.g., source codes to the Cyclops program [69,70] and in SwissSidechain database [65,66]. SMILES strings presented in the PubChem and ChemSpider databases do not maintain the above order.

**Figure 2:**
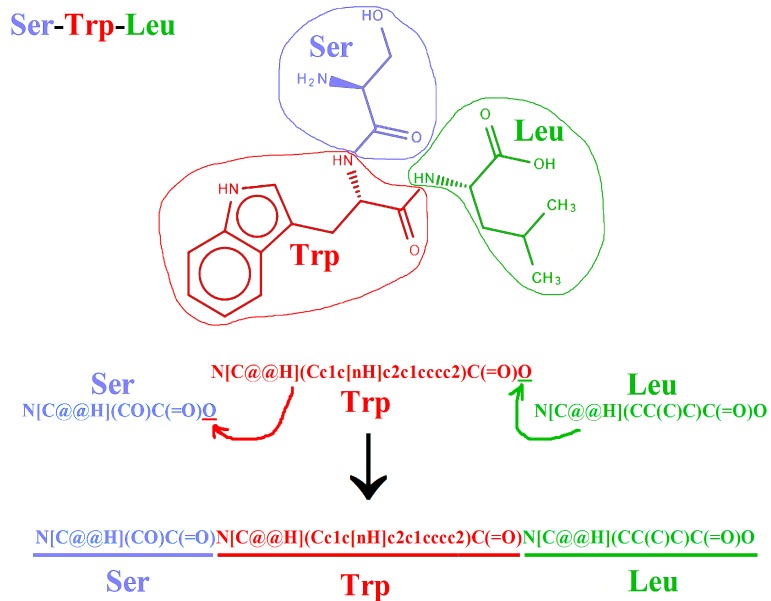
Scheme illustrating manual construction of SMILES representation of peptide SWL (Ser-Trp-Leu): Underlined oxygen atom in SMILES strings of individual amino acid molecues is replaced by the next amino acid residues during construction of peptide representation. Picture of peptide structure prepared with the help of Ketcher 2.0 molecular editor (Demo version).

Manual construction of SMILES representation of the exemplary tripeptide, based on recommendation of Siani and co-workers [2] is illustrated in Figure 2. The SMILES strings of an individual amino acids follow the order presented in Figure 1. The last oxygen atom in the carboxyl group (underlined in Figure 1 and Figure 2) may be replaced by the fragment that is corresponding to the next residue.

There are two freely accessible programs that are able to convert amino acid sequences of peptides into SMILES representations–downloadable program Open Babel [88,89] and CycloPs [69,70], available at its own server.

The Open Babel program (current version: 2.4.1) is able to translate peptide sequences annotated in a single-letter code into SMILES or other chemical codes and formats (recently 110 formats). The program utilizes FASTA format [90,91] as an input. The FASTA format is widely used for protein sequence annotation in, e.g., the UniProt database (European Bioinformatics Institute, Hinxton, UK) [92,93]. Conversion of peptide sequences from the FASTA format into SMILES is not described in the program manual, and requires a special correction procedure, as described in the supplement to our previous article [44]. Open Babel program utilizes sequences consisting of 20 common protein amino acids. The program is able also to convert peptide structures, annotated as pdb files [94] e.g., created by (PS)^2^. v3 or PepstrMod program. The second opportunity allows taking into account amino acid modifications, as accepted by the program. The order of symbols in the SMILES strings created by Open Babel differs from that created manually, as presented in Figure 2.

The CycloPs program utilizes common protein amino acids, as well as their D-enantiomers. The program applies an algorithm, as presented in Figure 2, to create SMILES representations of linear peptides. The creation of SMILES representations of cyclic peptides using CycloPs is also feasible. Another advantage of CycloPs as compared with Open Babel is its speed, especially in the case of processing sequences with length exceeding 10 amino acid residues.

Biomolecule toolkit (Provider: ChemAxon, Budapest, Hungary) is a commercially available resource for creation of biomolecule (e.g., peptide) annotations. It utilizes both peptide sequences (including non-natural and modified amino acids) and SMILES. This toolkit accepts peptides annotated using HELM notation [74,75] as an input.

### 2.3. Construction of SMILES Representations of Peptide Containing Modified Amino Acid Residues Using Glycosylated Amino Acids as an Example

Non-protein, non-natural, or modified amino acid residues may be inserted into SMILES representations of peptides via two ways. The first one includes the manual insertion of amino acid representations, which are taken from such databases as SwissSidechain. SMILES codes of amino acids, taken from PubChem, ChemSpider or ZINC 15 may need rearrangement to obtain the order of atom symbols presented in Figure 1. The second option is to apply molecular editors. They serve to display and modify structures of chemical compounds [95]. They may serve for implementation of simplified version of “forward translation flowchart” proposed by Siani and co-workers [2]. The editors sufficient to modify SMILES representations of peptides should provide the following opportunities: input and output of the molecular structure as a SMILES string, as well as display of the absolute configuration of substituents around asymmetric carbon atoms (Rectus or Sinister). Ketcher version 2.0 [86,87] and Marvin editor versions (Marvin Sketch, Marvin JS) (ChemAxon, Budapest, Hungary) [27] are examples of molecular editors that are fulfilling these requirements. The Marvin JS is widely used in chemical databases, e.g., ChEMBL [76,77] or HMDB [84,85].

**Figure 3:**
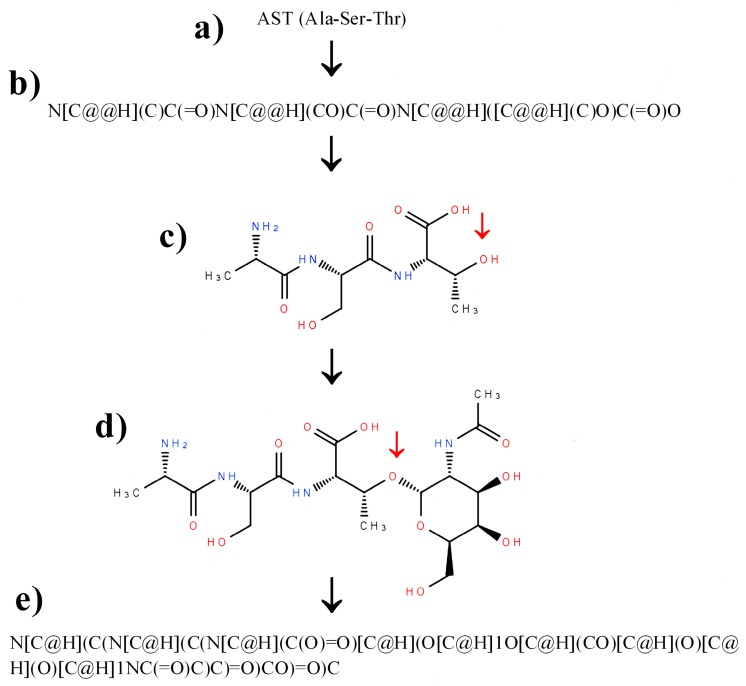
Scheme illustrating construction of SMILES representation of a modified peptide AST (Ala-Ser-Thr): (**a**) peptide sequence; (**b**) SMILES representation of unmodified peptide, constructed in agreement with scheme presented in Figure 2; (**c**) structure of unmodified peptide, displayed using program Ketcher (v. 2.0); and, (**d**) structure of peptide with a glycosylated threonine residue in position 3 (added residue of *N*-Acetyl-α-d-galactosamine–PubChem CID 84265); (**e**) SMILES representation of glycosylated peptide. Red arrows indicate modification site in structures of peptide and glycopeptide.

The scheme of inserting unnatural or modified amino acids into a peptide structure, annotated via the SMILES code with the help of molecular editor, may include the following steps: finding a protein amino acid or its enantiomer most similar to the desired one; construction of peptide SMILES representation using a selected amino acid residue; display and modification of a peptide structure using molecular editor; and, the conversion of the resulting, modified structure into SMILES. The above scheme is presented in Figure 3, with peptide containing a glycosylated threonine residue as an example. We use sugar moitety as an example of peptide modification for two reasons. The first one is fact that glycan residues are commonly present in peptides and proteins, and play very significant role in their biological activity [96,97,98]. The second reason is that sugar moieties are relatively complex as compared with other non-amino acid resides that are present in peptides. Someone who can correctly introduce sugar moiety may do that with other, less complex residue. The initial sequence of peptide includes an unmodified threonine residue as the most similar to the glycosylated threonine (Figure 3a). The second step (Figure 3b) involves the construction of SMILES representation of peptide (built manually or using appropriate software). The third step includes the import and display of the peptide structure in the molecular editor (Figure 3c). This step enables the verification of structure and the correction of errors in the peptide backbone structure (For details see Section 3). The fourth step (Figure 3d) includes building additional groups from the so-called “basic primitives” [99]—the simplest fragments of molecule structure that is used by molecular editors. If the additional group contains chirality centers, absolute configuration around asymmetric carbon atoms should be checked. The additional group (e.g., glycosidic, as shown in Figure 3d) usually mimics individual compound, which can be used as a reference molecule to check details of structure and chirality. In the example discussed herein, it is *N*-Acetyl-α-d-galactosamine (IUPAC name: *N*-[(2*S*,3*R*,4*R*,5*R*,6*R*)-2,4,5-trihydroxy-6-(hydroxymethyl)oxan-3-yl]acetamide; PubChem CID 84265).

**Figure 4:**
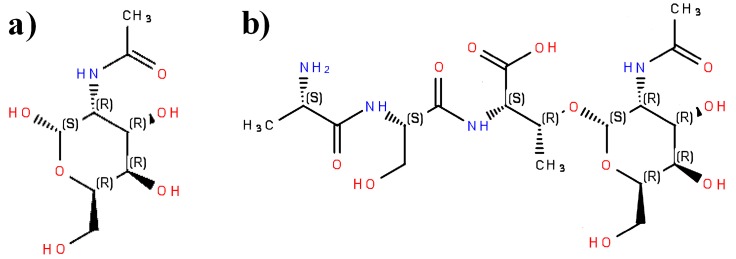
Structures of (**a**) *N*-Acetyl-α-d-galactosamine; (**b**) exemplary glycopeptide containing *N*-Acetyl-α-d-galactosamine residue with displayed absolute configuration of substituents around asymmetric carbon atoms. R—configuration Rectus; S—configuration Sinister. Structures displayed using Ketcher 2.0 program.

A comparison of absolute configurations around asymmetric carbon atoms in *N*-acetylgalactosamine and glycopeptide containing *N*-acetylgalactosamine residue is displayed in Figure 4. In the case of the compound presented above, the addition of the peptide chain does not affect configuration around asymmetric carbon atom being anomeric in Figure 4a and is involved in glycosidic bond in Figure 4b, in the sugar residue, as judged using Cahn-Ingold-Prelog priority rules [100,101]. The addition of the peptide chain does not change the priority of substituents around carbon atom from *N*-acetylgalactosamine residue, involved in the formation of *O*-glycosidic bond. Configuration “Sinister” is thus retained for this atom. Configuration (Rectus) remains the same also for carbon atom C3 in a threonine residue (See Figure 3c,d and Figure 4b). Change of the absolute configuration of asymmetric carbon atoms is, however, possible for other groups that are added to the peptide chain.

We have chosen the addition of a sugar residue as an example of peptide modification due to difficulties that are posed by the presence of several asymmetric carbon atoms in a single residue or to a discrepancy in numbering the carbon atoms. According to the rules that are accepted in sugar chemistry and glycobiology, the anomeric carbon atom in aldoses and their derivatives possesses number 1. Computer programs such Chemical Identifier Resolver (National Institutes of Health, Bethesda, MD, USA) [102,103] or Chemical Translation Service (University of California Davis, Davis, CA, USA) [104,105] use IUPAC rules for heterocyclic compounds for numbering carbon atoms in sugar rings. According to these rules, the anomeric carbon atom possesses number 2. IUPAC name of *N*-Acetyl-α-d-galactosamine, present in PubChem database (CID 84265), follows rules that are proposed for heterocyclic compounds. The introduction of many other modifications, such as C-terminal amidation, esterification, or charge of ionizable groups, is much easier.

Translation from the SMILES code into amino acid sequences of peptides is possible using the Smiles2Monomers program (University of Sciences and Technologies of Lille, Villeneuve d’Ascq, France) [106,107], which is associated with the Norine database [67,68]. This program utilizes SMILES strings of amino acid residues that are present in peptides annotated in the Norine database. Another, commercially available option for this purpose is Biomolecule Toolkit, provided by ChemAxon. This program utilizes chemical codes as an input via Marvin editor [27].

### 2.4. Other Chemical Codes

InChI and InChIKey [79] are another common formats applied to describe chemical compounds including peptides. In contrast to SMILES, inChI and InChIKey provide unique representation of a molecule structure. InChI code describes the structure of a molecule, whereas InChIKey, always containing 27 characters, does not reflect the molecule strucure, but may serve as a query for search using popular search engines, such as Google^TM^ [108,109]. In addition, InChIKeys are utilized by specialized programs for finding compounds in multiple databaes, such as Chemical Translation Service.

SMILES or InChI may be converted into many other formats using, e.g., OpenBabel program. Chemical formats are utilized, for instance, by programs designed to model interactions between proteins and small molecules. SwissDock (Swiss Institute of Bioinformatics, Lausanne, Switzerland) [110,111], AMMOS2 (Université Paris Diderot, Paris, France) [112,113], and ProteinsPlus (University of Hamburg, Germany) [114,115] are examples of such programs.

## 3. Verification and Correction of Errors in Peptide Structures Annotated Using SMILES Code

### 3.1. Typical Errors in Peptide Representations and Their Correction

**Figure 5:**
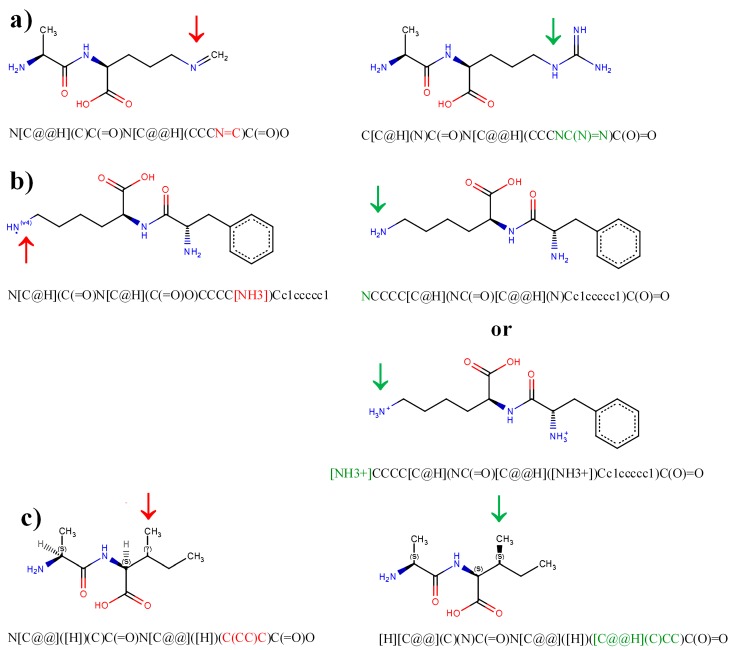
Structures of peptides displayed by molecular editor with errors (left column) and after correction (right column). (**a**) Peptide AR (Ala-Arg) with missed nitrogen atoms in guanidine group; (**b**) peptide FK (Phe-Lys) with inappropriate valence of nitrogen atom in side chain of lysine; (**c**) peptide AI (Ala-Ile) with missed chirality center in Ile residue. Red arrows and red fonts in SMILES representations indicate molecule fragments with errors. Green arrows and green fonts in SMILES representations indicate the same fragments after correction. R—configuration *Rectus*; S—configuration *Sinister*; ?—undefined configuration of substituents around asymmetric carbon atom. Figure prepared with the help of Marvin JS editor [27].

Characteristic errors in structures of peptides, as annotated using the SMILES code, are summarized in Table 1 and Figure 5. Inappropriate structure or configuration of a molecule fragment may lead to false negative results of database searching, or generate errors in prediction of physicochemical properties or interactions with biomacromolecules. Some of the errors may be detected and displayed automatically (Figure 5). The Marvin JS molecular editor is sufficient for this purpose. It displays inappropriate valence of atoms or missed chirality centers.

Missed atoms or inappropriate structures of functional groups (Figure 5a) are not detected automatically. In this case, the structures that are displayed reflect existing or at least stable molecules, but differ from the desired ones. The group indicated using red arrow in Figure 5a contains a nitrogen atom possessing valence 3, and a carbon atom with valence 4 (typical valences of both elements), but differs from true guanidine group in arginine residue (indicated using green arrow). Structures not possessing inappropriate valence or undefined chirality centers are accepted by the molecular editor and can be verified and corrected only manually—i.e., by deletion of the inappropriate fragment of a molecule and construction of an appropriate one from basic primitives. Guanidine group in Figure 5a has been corrected in this way.

Figure 5b presents an example of a structure containing atom with inappropriate valence. The example presents the ε-amine group of a lysine residue, containing nitrogen atom with valence 4 (–NH_3_ group without formal charge). This error may be important in the light of recommendations for possible molecular docking studies. Peptide structure input should include hydrogen atoms [88]. The error may be corrected by deletion of the inappropriate –NH_3_ group and insertion of an amine group –NH_2_, or a protonated amine group –NH_3_^+^ if necessary. Inappropriate valence is clearly shown in structures displayed using Marvin JS editor (Figure 5b, left column).

Missed chirality center is another kind of common error in compound structures. Configuration around asymmetric carbon atoms in molecules strongly affects interactions with biomacromolecules and hence biological activity. In the case of peptides, configuration around asymmetric carbon atom C3 in isoleucine or threonine residues should be taken into account. Peptide containing the isoleucine residue with missed chirality center at C3 carbon atom is presented in Figure 5c. Missed chirality center is displayed in InChI strings, which is converted from SMILES by the Open Babel or Marvin JS program, using “?” character. The same character is shown in the structure displayed using the second program (Figure 5c, left column). The structure should be corrected by insertion of an appropriate basic primitive [99] to achieve appropriate configuration around the chiral carbon atom. In L-isoleucine, the C3 carbon atom has *Sinister* (S) configuration, whereas in L-threonine—*Rectus* (R) configuration. In D enantiomers of these amino acids, the configuration around carbon atom C3 is reverse to the above. Another opportunity for error detection is Structure Checker application (provider. ChemAxon, Budapest, Hungary) [27].

### 3.2. Verification and Correction of Representations of Non-Peptidic Moiteties with Special Attention on Sugar Residues

The following additional procedure of verification of non-peptidic groups is possible if their structure corresponds to the known compounds that are present in general databases, such as PubChem. The SMILES code may be imported to the molecule editor. A peptide chain may be removed from the displayed structure. For instance, the removal of a peptide chain from the compound presented in Figure 4b provides the compound presented in Figure 4a. The resulting structure of non-peptidic moiety may be converted into SMILES and InChIKey, and may be used as a query in database searching (directly or via Chemical Translation Service). Finding compound in databases implies correctness of its structure. Apart from general databases such as PubChem or ChemSpider, Chemical Translation Service covers also LIPID MAPS^®^ (University of California Sand Diego, San Diego, CA, USA) [116,117]—a database designed for lipid annotation. It may serve for verification of lipid moieties in lipopeptides.

Verification of structures of sugar residues such as this presented in Figure 4, is possible also via specific tools designed for annotation and processing of carbohydrate structures. Databases of sugars and tools applied in glycoinformatics were reviewed by Campbell and co-workers [118]. WURCS program (WURCS Working Group, Japan) [119,120] and GlyTouCan database (Soka University, Tokyo, Japan) [121,122] may be helpful in the verification of correctness of simple carboydrate moieties. The protocol presented below may be used to this end.

Structures of sugar moiety and entire glycopeptide (See Figure 4a,b, respectively) should be converted into MDL molfile format [123]. Molecule editors, such as Ketcher 2.0 or Marvin JS, enable output of compound structure in MDL molfile format. OpenBabel program is also able to translate a molecule structure into this format. The structure converted into the above format may be transferred to the molecular editor at WURCS program website (“Chemical structure to WURCS” tab). This tab serves to translate sugar structure from MDL molfile format into WURCS code. The last one is a machine-readable code designed for description of carbohydrates [124]. The above-mentioned option results in the same WURCS representation of glycopeptide and its sugar moiety. For instance, *N*-acetyl-α-d-galactosamine (Figure 4a) and glycopeptide containing this sugar moiety (Figure 4b) possesses the following WURCS representation: WURCS=2.0/1,1,0/[a2112h-1a_1-5_2*NCC/3=O]/1/. This representation is identical also for *N*-acetyl-α-d-galactosamine structure taken from PubChem (CID 84265) and ChemSpider (ID 76020). The use more databases is recommended to avoid potential errors that are associated with, e.g., missed chirality centers in carbohydrate structures. WURCS representation may also be used as a query in GlyTouCan database screening using text search option. GlyTouCan accession number of *N*-acetyl-α-d-galactosamine is G57321FI. Finding sugar structure in GlyTouCan is a way to prove its correctness. The construction of glycan moiety of glycopeptide may be facilitated by the use of Marvin JS editor that is installed at the website of JCGGDB database [125,126]. The editor installed there offers additional option for construction of glycan moitey from building blocks (e.g., hexose ring, pentose ring etc.). Building blocks do not contain information concerning the configuration around particular asymmetric carbon atoms, which should be added manually.

Although time-consuming, the application of systematic procedures for verification and correction of molecular structures of compounds of interest (e.g., peptides) may allow avoiding errors in research conducted with the help of cheinformatic and bioinformatic methods. Alves and co-workers [127] have also pointed out the significance of transparency of cheminformatic procedures. Details of a procedure for verification and correction of compound structures that are used in a database or a dataset should be included in publications or their supplements. Readers would be thus able to apply and/or improve the workflow. Correction of peptide structures annotated in the BIOPEP database of sensory peptides and amino acids [45] may serve as an example of a procedure fulfilling this recommendation.

## 4. Brief Recommendations for Training

The importance of training in cheminformatics has recently been emphasized by Tetko and co-workers [128]. They pointed out that cheminformatics needs experience in two areas; chemistry and informatics. In the case of peptide science, the users of existing databases and/or software (such as authors of this article who are not informaticians) need skills in two areas: chemistry and biochemistry with molecular biology. Apart from skills in informatics, development of new software requires sufficient experience in the two above-mentioned areas. Recommendations below concern information from the area of chemistry, which is sufficient in the approach that is mentioned in this publication. Information from this area may be important for biologists using amino acid sequences and abbreviation-based codes in describing other biomolecules (sugars, nucleotides, and acylglycerols).

The checklist of skills includes training in use of a molecular editor, taking into account molecule drawing, input and output options, as well as strong and weak points. The second step involves knowledge of all details of peptide structures, including protein, non-protein and non-natural amino acids as well as modifications (e.g., sugar or lipid moieties). Special attention should be paid to the configuration of substituents around asymmetric carbon atoms. Some amino acids contain more than one chirality center (e.g., C3 atoms in isoleucine and threonine). Additional moieties may also contain asymmetric carbon atoms. Configuration around some asymmetric carbon atoms in biologically-active molecules, such as acylglycerols or sugars, may be described in different languages. IUPAC recommends the use of absolute configuration (R or S) in systematic names of chiral compounds. Attention should thus be paid to the translation of stereospecific numbering of acylglycerols (*sn*-1 and *sn*-3) or nomenclature of anomeric carbon atoms in sugar residues (α or β) into absolute configuration of asymmetric carbon atoms (atom *sn*-2 in acylglycerols or anomeric atoms in sugars). User of this software should recognize its strong and weak points, as well as the possible errors (e.g., generation of structures containing atoms with inappropriate valence, missed chirality centers or reversed configuration around asymmetric carbon atoms). In the case of using few programs, it is important to check co-operation between them.

The training scheme involves options and protocols that are described in the previous sections. The initial training dataset should contain simple compounds, mainly di- and tripeptides built from typical protein amino acids. They are usually annotated in general, chemical databases, such as PubChem, ChemSpider, and ChEMBL, and specialized peptide databases, such as BIOPEP or AHTPDB. Chemical codes that are generated during training may thus be easily verified. We confirm our previous recommendation [3] to try more databases. Simple di- and tripeptides are usually well described, but representations of more complex moieties (e.g., sugars) may contain errors. They may be recognized and removed via the confrontation of data from few databases. Sometimes, the creation of an appropriate structure representation requires a few attempts. For instance, any asymmetric carbon atom may occur in two possible configurations: R or S. The first attempt to obtain the correct one with the help of the molecular editor is successful with 50% likelihood (for one asymmetric carbon atom). The configuration of asymmetric carbon atoms is usually displayed in PubChem, or in names that are generated by Chemical Identifier Resolver, although for sugar moieties the numbering of carbon atoms, fulfilling IUPAC recommendations designed for heterocyclic compounds, may be confusing.

## 5. Final Remarks

A language barrier between biology and chemistry, or more precisely, between bioinformatics and cheminformatics, is a fact. Peptide science involves both approaches. Breaking this barrier would enhance progress in all research areas and disciplines interested in the structure and properties of peptides (e.g., medical, pharmaceutical, and food and nutritional sciences). The goal understood as correct and fluent translation from biological (amino acid sequences) into chemical (SMILES, InChI, etc.) is not achieved to date. Remarks and recommendations, presented herein, may help the users of databases and software working with sequences and structures of peptides to omit the language barrier. Delivery of new generation of translating software may provide opportunity to break this barrier in the area of peptides. The recommendations presented herein may be applied to develop datasets serving for testing such software. The applicability of some proposals presented here may also be extended to other classes of small biomolecules, such as sugars and lipids.

## Figures and Tables

**Table 1:** Possible errors in SMILES strings of peptides and their consequences.

Error	Consequences
Missed nitrogen atoms in guanidine groups	Possible errors in results of modeling interactions with biomacromolecules, inappropriate InChIKey, insufficient to be a query in database searching
Inappropriate valence of nitrogen atoms, e.g., in amine or guanidine groups	Possible errors in results of modeling interactions with biomacromolecules, unavailable for database search engines (e.g., for search engine of ZINC15 database), inappropriate InChIKey, insufficient to be a query in database searching
Undefined or inappropriate configuration of substituents around asymmetric carbon atom, e.g., C3 atom in isoleucine or threonine	Possible errors in results of modeling interactions with biomacromolecules, inappropriate InChIKey, insufficient to be a query in database searching
Spaces in SMILES strings	Disabled processing of SMILES strings by database search engines and other programs

## Abbreviations

ADME	Absorption, Distribution, Metabolism and Excretion
AHTPDB	Antihypertensive peptide database
CID	Compound Identifier (in PubChem database)
EMBL	European Molecular Biology Laboratory
α-GalNAc	*N*-Acetyl-α-d-galactosamine–PubChem CID 84265
HELM	Hierarchical Editing Language for Macromolecules
HMDB	Human Metabolome Database
InChI	International Chemical Identifier
InChIKey	Key of International Chemical Identifier
IUPAC	International Union of Pure and Applied Chemistry
LINUCS	Linear Notation for Unique Description of Carbohydrate Sequences
LIPID MAPS	Lipid Metabolites nd Pathways Strategy
MDL	Molecular Design Limited Inc. (company name)
QSAR	Quantitative Structure-Activity Relationship
R	One-letter symbol of amino acid arginine or absolute configuration of substituents around asymmetric carbon atom-*Rectus* (depending on context)
S	One-letter symbol of amino acid serine or absolute configuration of substituents around asymmetric carbon atom-*Sinister* (depending on context)
SATPdb	Structurally Annotated Therapeutic Peptide database
SMILES	Simplified Molecular Input Line Entry System or Simplified Molecular Input Line Entry Specification
WURCS	Web3 Unique Representation of Carbohydrate Structures
